# The dynamics of correlated novelties

**DOI:** 10.1038/srep05890

**Published:** 2014-07-31

**Authors:** F. Tria, V. Loreto, V. D. P. Servedio, S. H. Strogatz

**Affiliations:** 1 Institute for Scientific Interchange (ISI), Via Alassio 11C, 10126 Torino, Italy; 2 Sapienza University of Rome, Physics Dept., Piazzale Aldo Moro 5, 00185 Roma, Italy; 3 Institute for Complex Systems (ISC-CNR), Via dei Taurini 19, 00185 Roma, Italy; 4 Cornell University, Dept. of Mathematics, 310 Malott Hall, Ithaca, NY 14853, USA

## Abstract

Novelties are a familiar part of daily life. They are also fundamental to the evolution of biological systems, human society, and technology. By opening new possibilities, one novelty can pave the way for others in a process that Kauffman has called “expanding the adjacent possible”. The dynamics of correlated novelties, however, have yet to be quantified empirically or modeled mathematically. Here we propose a simple mathematical model that mimics the process of exploring a physical, biological, or conceptual space that enlarges whenever a novelty occurs. The model, a generalization of Polya's urn, predicts statistical laws for the rate at which novelties happen (Heaps' law) and for the probability distribution on the space explored (Zipf's law), as well as signatures of the process by which one novelty sets the stage for another. We test these predictions on four data sets of human activity: the edit events of Wikipedia pages, the emergence of tags in annotation systems, the sequence of words in texts, and listening to new songs in online music catalogues. By quantifying the dynamics of correlated novelties, our results provide a starting point for a deeper understanding of the adjacent possible and its role in biological, cultural, and technological evolution.

Our daily lives are spiced with little novelties. We hear a new song, taste a new food, learn a new word. Occasionally one of these first-time experiences sparks another, thus correlating an earlier novelty with a later one. Discovering a song that we like, for example, may prompt us to search for other music by the same artist or in the same style. Likewise, stumbling across a web page that we find intriguing may tempt us to explore some of its links.

The notion that one new thing sometimes triggers another is, of course, commonsensical. But it has never been documented quantitatively, to the best of our knowledge. In the world before the Internet, our encounters with mundane novelties, and the possible correlations between them, rarely left a trace. Now, however, with the availability of extensive longitudinal records of human activity online^1^, it has become possible to test whether everyday novelties crop up by chance alone, or whether one truly does pave the way for another.

The larger significance of these ideas has to do with their connection to Kauffman's theoretical concept of the “adjacent possible”^2^, which he originally discussed in his investigations of molecular and biological evolution, and which has also been applied to the study of innovation and technological evolution^3^,^4^. Loosely speaking, the adjacent possible consists of all those things (depending on the context, these could be ideas, molecules, genomes, technological products, etc.) that are one step away from what actually exists, and hence can arise from incremental modifications and recombinations of existing material. Whenever something new is created in this way, part of the formerly adjacent possible becomes actualized, and is therefore bounded in turn by a fresh adjacent possible. In this sense, every time a novelty occurs, the adjacent possible expands^5^. This is Kauffman's vision of how one new thing can ultimately lead to another. Unfortunately, it has not been clear how to extract testable predictions from it.

Our suggestion is that everyday novelties and their correlations allow one to test Kauffman's ideas quantitatively in a straightforward, down-to-earth setting. The intuition here is that novelties, like pre-biotic molecules and technological products, naturally form networks of meaningful associations. Just as a molecule in the primordial soup is conceptually adjacent to others that are one elementary reaction step away from it, a web page is conceptually adjacent to others on related topics. So when a novelty of any kind occurs, it does not occur alone. It comes with an entourage of surrounding possibilities, a cloud of other potentially new ideas or experiences that are thematically adjacent to it and hence can be triggered by it.

To avoid confusion, we should clarify a distinction we have in mind between novelties and innovations. We take an innovation to mean something created for the first time, something new to the world, something never seen before. A novelty, by contrast, is merely anything that is new to *you* (or to someone else). For example, a novelty could be a slang expression you have never heard before, or your first exposure to French New Wave cinema. That unfamiliar bit of slang and that Godard film already existed before you came upon them. They are therefore not innovations, but they count as novelties… to you. Seen in this light, an innovation is a very special sort of novelty. Innovations are novelties to *everyone*.

More important than this distinction, however, is what novelties and innovations share. Both form networks of meaningful associations, for the reasons discussed above; both come with entourages. Thus we are led to consider the possibility that all novelties, not just innovations, are subject to the correlations imposed by the expansion into the adjacent possible. If this hypothesis is borne out by the data, it would build a bridge between the well-established study of innovations in biological^6^,^7^,^8^,^9^, technological and social systems^10^,^11^,^12^,^13^,^14^,^15^ and the ubiquitous but often overlooked novelties we all experience every day. Indeed, as we will see below, although two of our data sets deal with innovations and two with novelties, all of them display the same three statistical patterns predicted by our model. These statistical commonalities support our hypothesis that novelties and innovations are two sides of the same dynamical coin.

## Results

### Human activities data

We begin by analyzing four data sets, each consisting of a sequence of elements ordered in time: (1) *Texts*: Here the elements are words. A novelty in this setting is defined to occur whenever a word appears for the first time in the text; (2) *Online music catalogues*: The elements are songs. A novelty occurs whenever a user listens either to a song or to an artist that she has not listened to before; (3) *Wikipedia*: The elements are individual wikipages. A novelty corresponds to the first edit action of a given wikipage by a given contributor (the edit can be the first ever, or other contributors may have edited the page previously but that particular contributor has not); (4) *Social annotation systems*: In the so-called tagging sites, the elements are tags (descriptive words assigned to photographs, files, bookmarks, or other pieces of information). A novelty corresponds either to the introduction of a brand new tag (a true innovation), or to its adoption by a given user. Further details on the data sets used are reported in the Supplementary Information.

**Figure 1:**
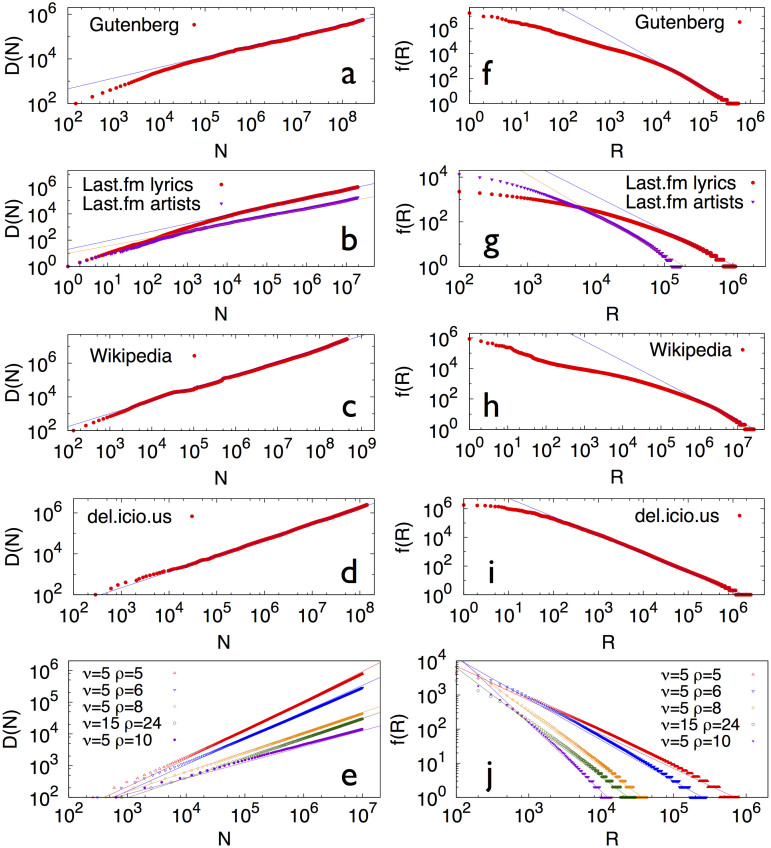
Heaps' law (a–e) and Zipf's law (f–l) in real datasets (a–d) and (f–i) and in the urn model with triggering (e,j). Gutenberg^42^ (a), (f), Last.fm^43^ (b), (g), Wikipedia^44^ (c), (h), del.icio.us^45^ (d), (i) datasets, and the urn model with triggering (e), (j). Straight lines in the Heaps' law plots show functions of the form *f*(*x*) = *ax^β^*, with the exponent *β* equal respectively to *β* = 0.45 (Gutenberg), *β* = 0.68 (Last.fm lyrics), *β* = 0.56 (Last.fm artist), *β* = 0.77 (Wikipedia) and *β* = 0.78 (del.icio.us), and to the ratio *ν*/*ρ* in the urn model with triggering, showing that the exponents for the Heaps' law of the model predicted by the analytic results are confirmed in the simulations. Straight lines in the Zipf's law plots show functions of the form *f*(*x*) = *ax*^−*α*^, where the exponent *α* is equal to *β*^−1^ for the different *β*'s considered above. Note that the frequency-rank plots in real data deviate from a pure power-law behavior and the correspondence between the *β* and *α* exponents is valid only asymptotically (see discussion above and the Supplementary Information for a discussion about finite-size effects).

The rate at which novelties occur can be quantified by focusing on the growth of the number *D*(*N*) of distinct elements (words, songs, wikipages, tags) in a temporally ordered sequence of data of length *N*. Figure 1 (a–d) shows a sublinear power-law growth of *D*(*N*) in all four data sets, each with its own exponent *β* < 1. This sublinear growth is the signature of Heaps' law^16^. It implies that the rate at which novelties occur decreases over time as *t^β^*^−1^.

A second statistical signature is given by the frequency of occurrence of the different elements inside each sequence of data. We look in particular at the frequency-rank distribution. In all cases (Fig. 1, f–i) the tail of the frequency-rank plot also follows an approximate power law (Zipf's law)^17^. Moreover, its exponent *α* is compatible with the measured exponent *β* of Heaps' law for the same data set, via the well-known relation *β* = 1/*α*^18^,^19^. It is important to observe that the frequency-rank plots are far from featuring a pure power-law behavior. In particular, the relation *β* = 1/*α* between the exponent *β* of Heaps' law and the exponent *α* of Zipf's law is expected to hold only in the tail of the Zipf plot. Moreover, the frequency-rank plots feature a variety of system-specific behaviors. For instance, for text corpora the frequency-rank plot features a 1/*R* trend for intermediate ranks (between 10 and 10^4^); a flattening of the slope for the most frequent words; and a larger slope, with an exponent compatible with the observed Heaps' law, for rare and specialized words. Reproducing such features quantitatively would require a more detailed modeling scheme than we consider here, including for instance the distinctions between articles, prepositions, and nouns. Nevertheless it is interesting that our model with semantic triggering predicts a double slope for Zipf's law^20^ as a consequence of the correlations induced by the parameter *η* (see Supplementary Information for further details).

Next we examine the four data sets for a more direct form of evidence of correlations between novelties. To do so we need to introduce the notion of semantics, defined here as meaningful thematic relationships between elements. We can then consider semantic groups as groups of elements related by common properties. The actual definition of semantic groups depends on the data we are studying, and can be straightforward in some cases and ambiguous in others. For instance, in the Wikipedia database, we can regard different pages as belonging to the same semantic group if they were created for the first time linked to the same mother page (see Supplementary Information for further details). In the case of the music database (Last.fm), different semantic groups for the listened songs can be identified with the corresponding song writers. In the case of texts or tags, there is no direct access to semantics, and we adopted a slightly different procedure to detect semantically charged triggering events. Lacking of a satisfactory classification of words in semantic areas, for text and tags we considered each word/tag as bearing its own class.

Also in these cases the triggering of novelties can be observed by looking at the highly nontrivial distribution of words. We refer to the Supplementary Information for a detailed discussion of these cases.

We now introduce two specific observables: the entropy *S* of the events associated to a given semantic group, and the distribution of time intervals *f*(*l*) between two successive appearances of events belonging to the same semantic group. Roughly speaking, both the entropy *S* and the distribution of time intervals *f*(*l*) measure the extent of clustering among the events associated to a given semantic group, with a larger clustering denoting stronger correlations among their occurrences and thus a stronger triggering effect (refer to the Methods section).

**Figure 2:**
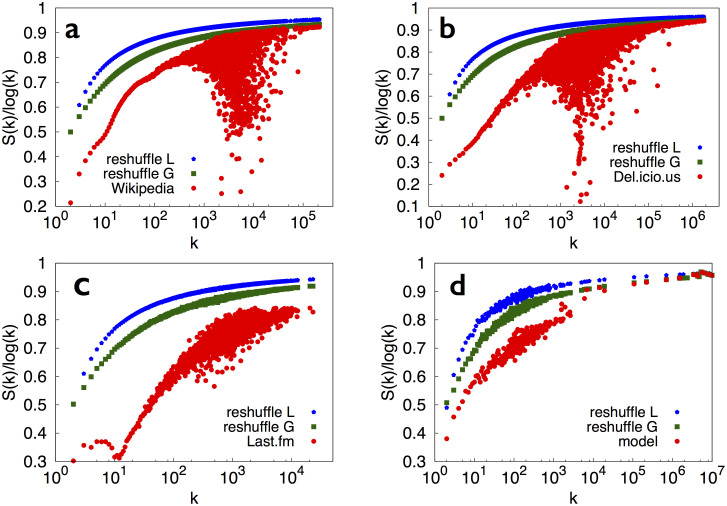
Normalized entropy in real data and in the urn model with semantic triggering. (a), (b), (c) Normalized entropy of a sequence associated to a specific label *A* vs. the number of events, *k*, with that label. The entropy is averaged for each *k* over the labels with the same number of occurrences. Results are displayed for Wikipedia (a), the Delicious dataset (b), the Last.fm dataset (c) and the urn model with semantic triggering (d). For the Wikipedia and Last.fm datasets we used the respective sequences *S*_unique_ as described in the section Methods, while for the Delicious dataset we used the full sequence of aggregated data. The plot for the model is an average over 10 realizations of the process, with parameters *ρ* = 8, *ν* = 10, *η* = 0.3 and *N*_0_ = *ν* + 1. The length of the considered sequences is *N* = 10^7^ and the corresponding Heaps' exponent is 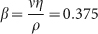 (see Supplementary Information for the relation of the Heaps' and Zipf's exponents with the model parameters). In all the cases, results for the actual data are compared with two null models, as described in the section Methods.

All the data sets display the predicted correlations among novelties. The results for the entropy in Wikipedia, the social annotation system del.icio.us and Last.fm databases are shown in Fig. 2 (a,b,c). For comparison, we also reshuffle all the data sets randomly to assess the level of temporal correlations that could exist by chance alone (refer to the Methods section for details). The evidence for semantic correlations is signaled by a drop of the entropy *S* with respect to the reshuffled cases in all the databases considered (Fig. 2, a, b, and c).

**Figure 3:**
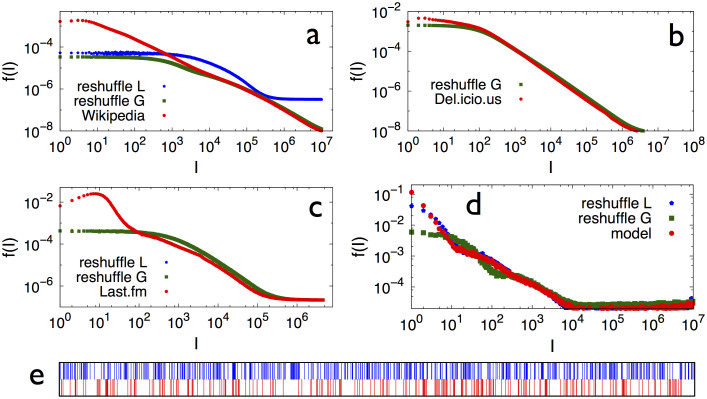
Distribution of triggering intervals in real data and in the urn model with semantic triggering. Results for the distribution of triggering intervals (see the section Methods for the definition) for the same data as for the entropy measurements in figure 2: Wikipedia (a), the Delicious dataset (here the calculation of the local reshuffling was too time-consuming due to the fact that there are as many labels as words), (b), the Last.fm dataset (c) and the urn model with semantic triggering (d). The banner at the bottom(e) shows a 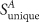 sequence for a particular label *A* of the Last.fm dataset. The color code is red for the actual sequence 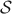 and blue for the local reshuffle (see methods section) of 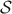.

The distributions *f*(*l*), reported in Fig. 3 (a,b,c) for Wikipedia, del.icio.us and Last.fm, respectively, feature a markedly larger peak for short time intervals compared to that seen in the random case, indicating that events belonging to the same semantic group are clustered in time (Fig. 3e).

**Figure 4:**
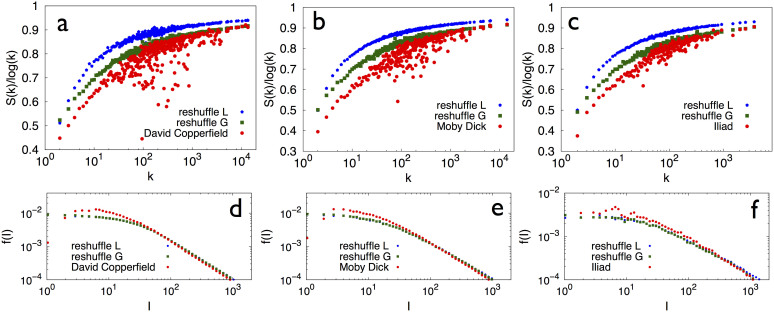
Normalized entropy and distribution of triggering intervals in single books of the Gutenberg dataset. (a), (b), (c) Normalized entropy of words (as described in the main text) vs. the number of words occurrence, *k*. The entropy is averaged for each *k* over the words with the same number of occurrences. Results are displayed for the texts David Copperfield (a), Moby Dick (b) and Iliad (in the original greek version) (c). (d), (e), (f) Results for the distribution of triggering intervals for the same data as above. In all the cases, results for the actual data are compared with the two null models described in the section Methods.

It is interesting to observe that Wikipedia, del.icio.us and Last.fm represent the outcome of a collective activity of many users. A natural question is whether the correlations observed above only emerge at a collective level or are also present at an individual level. In order to investigate this point we focus on individual texts (i.e., written by single authors of the Gutenberg corpus (see Methods section for details). Fig. 4 (a,b,c) report both the normalized average entropies in selected texts (red dot) and in the locally (blue dots) and globally (green dots) reshuffled texts. Also in this case lower values of the entropy correspond to more highly clustered occurrences of elements. Fig. 4 (d,e,f) report the time intervals distribution *f*(*l*) for the same set of texts and reshuffled sets. More highly clustered data result in higher values of the distribution at low interval lengths. It is interesting to observe how each individual (each author in this case) reproduces the qualitative features of aggregated data (analyzed for the three other datasets): namely, a significantly higher clustering than that found in the reshuffled data. We refer to the Supplementary Information for a more extensive analysis performed for single users in the different databases, confirming the same qualitative scenario. The above reported results show that the adjacent possible mechanism plays a role also on the individual level, and its effect is enhanced in collective processes.

### A simple generative model

Our results so far are consistent with the presence of the hypothesized adjacent possible mechanism. However, since we only have access to the actual events and not to the whole space of possibilities opened up by each novelty, we can only consider indirect measures of the adjacent possible, such as the entropy and the distribution of time intervals discussed above.

To extract sharper predictions from the mechanism of an ever-expanding adjacent possible, it helps to consider a simplified mathematical model based on Polya's urn^21^,^22^,^23^. In the classical version of this model^21^, balls of various colors are placed in an urn. A ball is withdrawn at random, inspected, and placed back in the urn along with a certain number of new balls of the same color, thereby increasing that color's likelihood of being drawn again in later rounds. The resulting “rich-get-richer” dynamics leads to skewed distributions^24^,^25^ and has been used to model the emergence of power laws and related heavy-tailed phenomena in fields ranging from genetics and epidemiology to linguistics and computer science^26^,^27^,^28^.

This model is particularly suitable to our problem since it considers two spaces evolving in parallel: we can think of the urn as the space of possibilities, while the sequence of balls that are withdrawn is the history that is actually realized.

We generalize the urn model to allow for novelties to occur and to trigger further novelties. Our approach thus builds on that of Hoppe^29^ and other researchers (see Refs. 30, 31 and references therein), who introduced novelties within the framework of Polya's urn but did not posit that they could trigger subsequent novelties. (The problem of modeling novelties is very old and dates back to the work of the logician Augustus de Morgan^32^. For a review of this early work, see Ref. 33.). Hoppe's model was motivated by the biological phenomenon of neutral evolution, with novel alleles represented as an open-ended set of colors arising via mutation from a single fixed color. This variant of Polya's urn implies a logarithmic, rather than power-law, form for the growth of new colors in the urn, and hence does not account for Heaps' law. Hoppe's urn scheme is non-cooperative in the sense that no conditional appearance of new colors is taken into account; in particular, one novelty does nothing to facilitate another.

**Figure 5:**
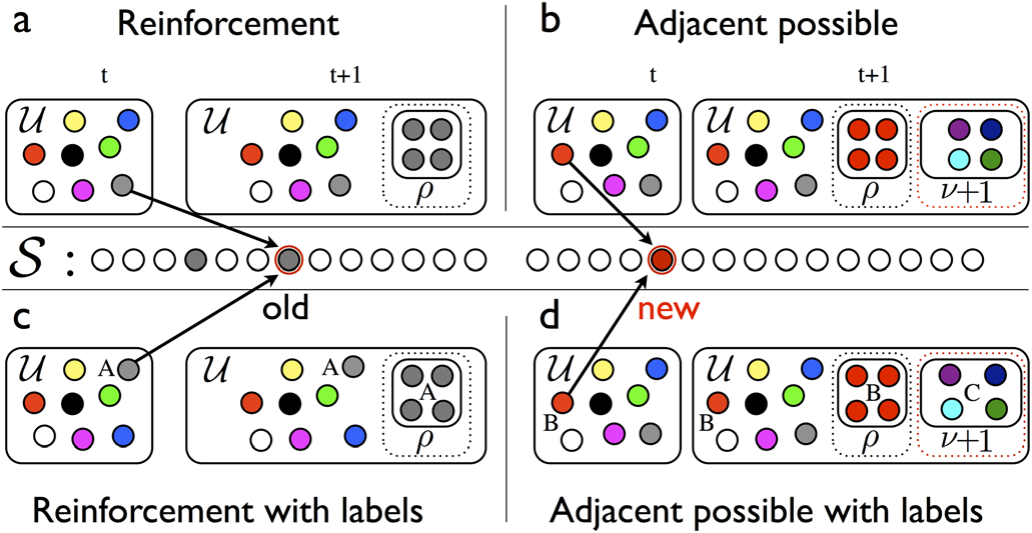
Models. Simple urn model with triggering (a), (b) and urn model with semantic triggering (c), (d). (a) Generic reinforcement step of the evolution. An element (the gray ball) that had previously been drawn from the urn 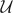 is drawn again. In this case one adds this element to 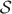 (depicted at the center of the figure) and, at the same time, puts *ρ* additional gray balls into 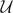. (b) Generic adjacent possible step of the evolution. Here, upon drawing a new ball (red) from 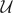, *ν* + 1 brand new balls are added to 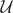 along with the *ρ* red balls of the reinforcement step that takes place at each time step. (c), (d) Urn model with semantic triggering. Same as above except that now each ball has a label defining its semantic context. The label is conserved during a reinforcement event (e.g., the label *A* for the gray balls on panel c) while it appears as a brand new label, *C*, for the *ν* + 1 balls added for an adjacent possible event (panel d).

In contrast, the cooperative triggering of novelties is essential to our model. Consider an urn 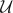 containing *N*_0_ distinct elements, represented by balls of different colors (Fig. 5). These elements represent words used in a conversation, songs we've listened to, web pages we've visited, inventions, ideas, or any other human experiences or products of human creativity. A conversation, a text, or a series of inventions is idealized in this framework as a sequence 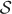 of elements generated through successive extractions from the urn. Just as the adjacent possible expands when something novel occurs, the contents of the urn itself are assumed to enlarge whenever a novel (never extracted before) element is withdrawn.

Specifically, the evolution proceeds according to the following scheme. At each time step *t* we select an element *s_t_* at random from 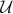 and record it in the sequence. We then put the element *s_t_* back into 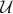 along with *ρ* additional copies of itself. The parameter *ρ* represents a reinforcement process, i.e., the more likely use of an element in a given context. For instance, in a conversational or textual setting, a topic related to *s_t_* may require many copies of *s_t_* for further discussion. The key assumption concerns what happens if (and only if) the chosen element *s_t_* happens to be novel (i.e., it is appearing for the first time in the sequence 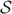). In that case we put *ν* + 1 brand new and distinct elements in the urn. These new elements represent the set of new possibilities triggered by the novelty *s_t_*. Hence *ν* + 1 is the size of the new adjacent possible made available once we have a novel experience. The growth of the number of elements in the urn, conditioned on the occurrence of a novelty, is the crucial ingredient modeling the expansion of the adjacent possible.

This minimal model simultaneously yields the counterparts of Zipf's law (for the frequency distribution of distinct elements) and Heaps' law (for the sublinear growth of the number of unique elements as a function of the total number of elements). In particular, we find that the balance between reinforcement of old elements and triggering of new elements affects the predictions for Heaps' and Zipf's law. A sublinear growth for *D*(*N*) emerges when reinforcement is stronger than triggering, while a linear growth is observed when triggering outweighs reinforcement. More precisely the following asymptotic behaviors are found (see Supplementary Information for the analytical treatment of the model): 
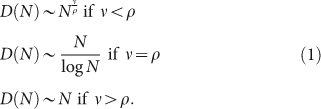
Correspondingly, the following asymptotic form is obtained for Zipf's law: 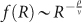, where *f*(*R*) is the frequency of occurrence of the element of rank *R* inside the sequence 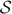. Figure 1 also shows the numerical results as observed in our model for the growth of the number of distinct elements *D*(*N*) (Fig. 1e) and for the frequency-rank distribution (Fig. 1j), confirming the analytical predictions.

### A model with semantics

So far we have shown how our simple urn model with triggering can account simultaneously for the emergence of both Heaps' and Zipf's law. This is a very interesting result *per se* because it offers a possible solution to the longstanding problem of explaining the origin of Heaps' and Zipf's laws through the same basic microscopic mechanism, without the need of hypothesizing one of them to deduce the other. (A different mechanism that yields both Zipf's law and Heaps' law has been proposed recently in a linguistic context by Gerlach and Altmann^20^.) Despite the interest of this result, this is not yet enough to account for the adjacent possible mechanism revealed in real data. In its present form, the model accounts for the opening of new perspectives triggered by a novelty, but does not contain any bias towards the actual realization of these new possibilities.

To account for this, we need to infuse the earlier notion of semantics into our model. We endow each element with a label, representing its semantic group, and we allow for the emergence of dynamical correlations between semantically related elements. The process we now consider starts with an urn 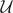 with *N*_0_ distinct elements, divided into *N*_0_/(*ν* + 1) groups. The elements in the same group share a common label. To construct the sequence 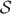, we randomly choose the first element. Then at each time step *t*, (i) we give a weight 1 to: (a) each element in 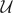 with the same label, say *A*, as *s_t_*_−1_, (b) to the element that triggered the entry into the urn of the elements with label *A*, and (c) to the elements triggered by *s_t_*_−1_. A weight *η* ≤ 1 is assigned to all the other elements in 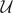. We then choose an element *s_t_* from 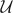 with a probability proportional to its weight and write it in the sequence; (ii) we put the element *s_t_* back in 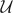 along with *ρ* additional copies of it (Fig. 5c); (iii) if (and only if) the chosen element *s_t_* is new (i.e., it appears for the first time in the sequence 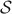) we put *ν* + 1 brand new distinct elements into 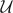, all with a common brand new label (Fig. 5d). Note that for *η* = 1 this model reduces to the simple urn model with triggering introduced earlier.

This extended model can again reproduce both Heaps' and Zipf's laws (for details, see the Supplementary Information), and it also captures some of the main qualitative features of *S* and *f*(*l*) seen in real data (Fig. 2, d and Fig. 3, d). Thus, the hypothesized mechanism of a relentlessly expanding adjacent possible is consistent with the dynamics of correlated novelties, at least for the various techno-social systems^34^ studied here.

## Discussion and Conclusions

Let us return to the question of whether novelties and innovations share the same dynamics. All four of our datasets displayed the same statistical patterns, both for the rates at which novel events occur and for the statistical signatures of triggering events. Two of the data sets involved innovations (the creation of brand new pages in Wikipedia and the introduction of brand new tags in del.icio.us), while the other two involved novelties that do not qualify as innovations (the first appearance of a word in a text, or the first time a user listens to an existing song). The fact that we observe the same statistical signatures for novelties and innovations strengthens the hypothesis that they could be two sides of the same coin, namely, manifestations of correlations generated by the expansion of the adjacent possible.

From this perspective we speculate that our theoretical framework could be relevant to a much wider class of systems, problems, and issues—indeed, to any situation where one novelty fosters another. A natural next step would be to focus more specifically on the study of major innovations in cultural^35^, technological^4^,^15^, and biological systems^6^,^8^,^36^. A huge literature exists on different aspects of innovation, concerning both its adoption and diffusion^37^,^38^,^39^,^40^, as well as the creative processes through which it is generated^6^,^7^,^10^,^14^. The deliberately simplified framework we have developed here does not attempt to model explicitly the processes leading to innovations^4^, such as recombination^10^,^14^, tinkering^6^ or exaptation^7^. Rather, our focus is entirely on the implications of the new possibilities that a novelty opens up. In our modeling scheme, processes such as the modification or recombination of existing material take place in a black box; we account for them in an implicit way through the notions of triggering and semantic relations. Building a more fine-grained mathematical model of these creative processes remains an important open problem.

Another direction worth pursuing concerns the tight connection between innovation and semantic relations. In preliminary work, we have begun investigating this question by mathematically reframing our urn model as a random walk. As we go about our lives, in fact, we silently move along physical, conceptual, biological or technological spaces, mostly retracing well-worn paths, but every so often stepping somewhere new, and in the process, breaking through to a new piece of the space. This scenario gets instantiated in our mathematical framework. Our urn model with triggering, in fact, both with and without semantics, can be mapped onto the problem of a random walker exploring an evolving graph 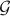. The idea of the construction of a sequence of actions or elements as a path of a random walker in a particular space has been already studied in Ref. 41, where it has been shown that the process of social annotation can be viewed as a collective but uncoordinated exploration of an underlying semantic space. One can go a step further by considering a random walker as wandering on a growing graph 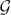, whose structure is self-consistently shaped by the innovation process, the semantics being encoded in the graph structure. This picture strengthens the correspondence between the appearance of correlated novelties and the notion of the adjacent possible. Moreover, this framework allows one to relate quantitatively, and in a more natural way, the particular form of the exploration process (modulated by the growing graph topology) and the observed outcomes of observables related to triggering events. We refer to the Supplementary Information for a detailed discussion of this mapping and results concerning this random-walk framework for the dynamics of correlated novelties.

Two more questions for future study include an exploration of the subtle link between the early adoption of an innovation and its large-scale spreading, and the interplay between individual and collective phenomena where innovation takes place. The latter question is relevant for instance to elucidate why overly large innovative leaps cannot succeed at the population level. On a related theme, the notion of advance into the adjacent possible sets its own natural limits on innovations, since it implies that innovations too far ahead of their time, i.e., not adjacent to the current reality, cannot take hold. For example, video sharing on the Internet was not possible in the days when connection speeds were 14.4 kbits per second. Quantifying, formalizing, and testing these ideas against real data, however, remains a fascinating challenge.

## Methods

### Data sets

Our analysis takes into account four different data sets. (i) The corpus of English texts consists of the material available at the Gutenberg Project ebook collection^42^ till February 2007 and resulted in a set of about 4600 non-copyrighted ebooks dealing with diverse subjects and including both prose and poetry. In total, the corpus consisted of about 2.8 × 10^8^ words, with about 5.5 × 10^5^ different words. In the analysis we ignored capitalization. Words sharing the same lexical root were considered as different, i.e., the word *tree* was considered different from *trees*. Homonyms, as for example the verbal past perfect *saw* and the substantive *saw*, were treated as the same word. (ii) Delicious^45^ is an online social annotation platform of bookmarking where users associate keywords (tags) to web resources (URLs) in a post, in order to ease the process of their retrieval. The dataset consists of approximately 5 × 10^6^ posts, comprising about 650,000 users, 1.9 × 10^6^ resources and 2.5 × 10^6^ distinct tags (for a total of about 1.4 × 10^8^ tags), and covering almost 3 years of user activity, from early 2004 up to November 2006. (iii) Last.fm^43^ is a music website equipped with a music recommender system. Last.fm builds a detailed profile of each user's musical taste by recording details of the songs the user listens to, either from Internet radio stations, or the user's computer or many portable music devices. The data set we used^43^,^46^ contains the whole listening habits of 1000 users till May, 5th 2009, recorded in plain text form. It contains about 1.9 × 10^7^ listened tracks with information on user, time stamp, artist, track-id and track name. (iv) The Wikipedia database we collected^44^ dates back to March 7th, 2012 and contains a copy of all pages with all their edits in plain text. Please refer to the Supplementary Information for a more detailed discussion of these data sets.

### Detecting triggering events

As pointed out above, the semantics and the notion of meaning could trigger non-trivial correlations in the sequence of words of a text, the sequence of songs listened to, or the sequence of ideas in a given context. In order to take into account semantic groups, we introduce suitable labels to be attached to each element of the sequence. For instance, in the case of music, one can imagine that when we first discover an artist or a composer that we like, we shall want to learn more about his or her work. This in turn can stimulate us to listen to other songs by the same artist. Thus, the label attached to a song would be, in this case, its corresponding writer.

**Figure 6:**
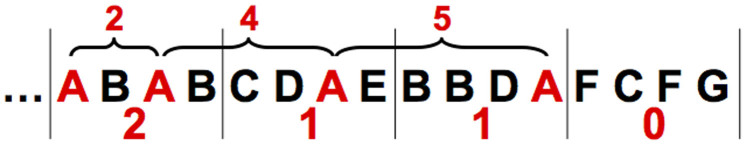
Entropy and intervals example. Let us indicate with the same letters the occurrences, e.g., of lyrics of the same artist in the sequence. Suppose that A has just appeared in the sequence, which ends with G. Thus, A appears 4 times, i.e., *k* = 4. We divide the subsequence 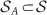 in 4 parts and count the occurrences *f_i_* of A in each of them (bottom numbers). The normalized entropy of A will be 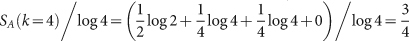. As a value of *S*(*k*) we average all entropies of the elements occurring *k*-times in 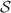. The numbers at the top show the length of the inter-times used in the interval distribution evaluation. The *local* reshuffling would shuffle only those 15 elements occurring after the first occurrence of A, and compute the normalized entropy and the time intervals distribution on this reduced sequence.

To detect such non-trivial correlations we define the entropy *S_A_*(*k*) of the sequence of occurrences of a specific label *A* in the whole sequence 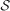, as a function of the number *k* of occurrences of *A*. To this end we identify the sub-sequence 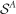 of 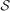 starting at the first occurrence of *A*. We divide 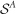 in *k* equal intervals and call *f_i_* the number of occurrences of the label *A* in the *i*-th interval (see Fig. 6). The entropy of *A* is defined as 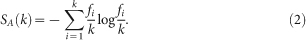
In case the occurrences of *A* were equally distributed among these intervals, i.e., *f_i_* = 1 ∀*i* = 1 … *k*, *S_A_*(*k*) would get its maximum value log *k*. On the contrary, if all the occurrences of *A* were in the first chunk, i.e., *f*_1_ = *k* and *f_i_*_≠1_ = 0, the entropy would get its minimum value *S_A_*(*k*) = 0. Each *S_A_*(*k*) is normalized with the factor 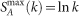, the theoretical entropy for a uniform distribution of the *k* occurrences. The entropy *S*(*k*) is calculated by averaging the entropies relative to those elements occurring *k*-times in the sequence.

Moreover, we also analyze the distribution of triggering time intervals *f*(*l*). For each label, say *A*, we consider the time intervals between successive occurrences of *A*. We then find the distribution of time intervals related to all the labels appearing in the sequence 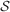 (see also Fig. 6).

In the Wikipedia and Last.fm datasets we can go a step further since they contain the contribution of many users. In this case we can focus on a sub-sequence 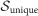 of 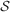 that neglects the multiple occurrence of the same element by the same users, e.g., in Last.fm multiple listening of the same song by the same users (a specific song can be present anyway several times in the sub-sequence since that song can be listened for the first time by different users). We can thus identify for each label, say *A*, the sub-sequence 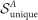 and correspondingly define the entropy and the time intervals distribution as described above (see also the Supplementary Information for a detailed discussion of this analysis both for Last.fm and Wikipedia).

### Reshuffling methods

In order to ground the results obtained, both for the entropy and the distribution of triggering intervals, we consider two suitably defined ways of removing correlation in a sequence. Firstly, we just *globally* reshuffle the entire sequence 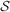. In this way semantic correlations are disrupted but statistical correlations related to the nonstationarity of the model (responsible, for instance, for Heaps' and Zipf's law) are still present. Secondly, for each label, we reshuffle the sequence 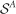 *locally*, i.e., from the first appearance of *A* onwards. This latter procedure removes altogether any correlations between the appearance of elements.

## Supplementary Material

Supplementary Information Supplementary Info
