# Supplementary material for: The dynamics of correlated novelties
Source: Sci Rep. 2014 Jul 31;4:5890. doi: 10.1038/srep05890 (PMC5376195; doi:10.1038/srep05890)
Supplement: Supplementary Information — Supplementary Info [file srep05890-s1.pdf]

The dynamics of correlated novelties

Supplementary Information

F. Tria[1], V. Loreto[2,1], V.D.P. Servedio[2,3] and S.H. Strogatz[4]

[1]Institute for Scientific Interchange (ISI), Viale Settimio Severo 65, 10133 Torino, Italy
[2]Sapienza University of Rome, Physics Dept., P.le Aldo Moro 5, 00185 Roma, Italy
[3]Institute for Complex Systems (ISC-CNR), Via dei Taurini 19, 00185 Roma, Italy
[4]Cornell University, Dept. of Mathematics, 310 Malott Hall, Ithaca, NY 14853, USA

1 Urn model with triggering

1.1 Model definition

In the main text we introduced the *urn model with triggering*. Briefly, an ordered sequence $\mathcal{S}$ was constructed by picking elements (or balls) from a reservoir (or urn) $\mathcal{U}$ initially containing N_0 distinct elements. Both the reservoir and the sequence increased their size according to the following procedure. At each time step:

(i) an element is randomly extracted from $\mathcal{U}$ with uniform probability and added to $\mathcal{S}$;

(ii) the extracted element is put back into $\mathcal{U}$ together with ρ copies of it;

(iii) if the extracted element has never been used before in $\mathcal{S}$ (it is a new element in this respect), then $\nu + 1$ different brand new distinct elements are added to $\mathcal{U}$.

Note that the number of elements N of $\mathcal{S}$, i.e. the length $|\mathcal{S}|$ of the sequence, equals the number of times t we repeated the above procedure. If we let D denote the number of distinct elements that appear in $\mathcal{S}$, then the total number of elements in the reservoir after t steps is $|\mathcal{U}|_t = N_0 + (\nu + 1)D + \rho t$.
In the following, we shall also consider a second and slightly different version of

the model, in which the reinforcement does not act when an element is chosen for the first time. Hence, point (ii) of the previous rules will be changed into:

(ii.a) the extracted element is put back in $\mathcal{U}$ together with ρ copies of it *only if it is not new in the sequence*.

1.2 Computation of the asymptotic Heaps' and Zipf's laws

We discuss here the asymptotic behaviour of both the number of distinct elements $D(t)$ appearing in the sequence and the frequency-rank distribution $f(R)$ of the elements in the sequence $\mathcal{S}$. We will show that both versions of the urn model above predict a Heaps' law for $D(t)$ and a frequency-rank distribution $f(R)$ with a fat-tail behavior. Our calculations yield simple formulas for the Heaps' law exponent and the exponent of the asymptotic power-law behavior of the frequency-rank distribution in terms of the model parameters ρ and ν.

Strictly speaking, Zipf's law requires an inverse proportionality between the frequency and rank of the considered quantities [1]. In the following, however, we shall always refer instead to a generalized version of Zipf's law, in which the dependence of the frequency on the rank is power-law-like in the tail of the distribution, i.e. at large ranks.

Heaps' law

In the first version of the model, the time dependence of the number D of different elements in the sequence $\mathcal{S}$ obeys the following differential equation:

$$\frac{dD}{dt} = \frac{U_D(t)}{U(t)} = \frac{N_0 + \nu D}{N_0 + (\nu+1)D + \rho t}, \tag{1}$$

where $U_D(t)$ is the number of elements in the reservoir that at time t have not yet appeared in $\mathcal{S}$, and $U(t) = |\mathcal{U}|_t$ is the total number of elements in the reservoir at time t. The term νD in the numerator of the rightmost expression comes from the fact that each time a new element is introduced in the sequence, $U_D(t)$ is increased by ν elements (since $\nu + 1$ brand new elements are added to $\mathcal{U}$, while the chosen element is no longer new). Due to the inherently discrete character of D and t, Eq. (1) is valid asymptotically for large values of D and t.

In the second version of the model, Eq. (1) has to be modified by replacing the denominator with

$$U(t) = N_0 + (\nu+1)D + \rho(t-D) = N_0 + (\nu+1-\rho)D + \rho t.$$

To analyze both versions of the model simultaneously, it is convenient to define a parameter $a \equiv \nu + 1$ for the first version and $a \equiv \nu + 1 - \rho$ for the second version.

In order to obtain an analytically solvable equation, and since we are interested in the behaviour at large times $t \gg N_0$, we approximate equation (1) by

$$\frac{dD}{dt} = \frac{\nu D}{aD + \rho t}. \tag{2}$$

By introducing the auxiliary variable $z = \frac{D}{t}$ and performing some straightforward algebra we obtain the asymptotic behaviour of $D(t)$ for large t:

1. $\rho > \nu$: $D \sim (\rho - \nu)^{\frac{\nu}{\rho}} t^{\frac{\nu}{\rho}}$;

2. $\rho < \nu$: $D \sim \frac{\nu - \rho}{a} t$;

3. $\rho = \nu$: $D \log D \sim \frac{\nu}{a} t \rightarrow D \sim \frac{\nu}{a} \frac{t}{\log t}$,

For completeness, we note that both versions of the model can be regarded as the coarse-grained equivalent of a two-color asymmetric Polya urn model [2]. In particular, within that finer framework the substitution matrices (denoted M_1 for the first version of the model and M_2 for the second) would be:

$$M_1 = \begin{pmatrix} \rho & 0 \\ 1 + \rho & \nu \end{pmatrix} \text{ and } M_2 = \begin{pmatrix} \rho & 0 \\ 1 & \nu \end{pmatrix}.$$

In this interpretation, the elements that have already appeared in $\mathcal{S}$ are represented by balls of one color, while those that have not appeared yet correspond to balls of the other color.

Zipf's law

Making the same approximations as above, the continuous dynamical equation for the number of occurrences n_i of an element i in the sequence $\mathcal{S}$ can be written as

$$\frac{dn_i}{dt} = \frac{n_i \rho + 1}{N_0 + aD + \rho t}. \tag{3}$$

Two cases can be distinguished:

1. $\nu \leq \rho$, when $\lim_{t \rightarrow +\infty} D/t = 0$. By considering only the leading term for $t \rightarrow +\infty$, one has

$$\frac{dn_i}{dt} \simeq \frac{n_i}{t}. \tag{4}$$

Let t_i denote the time at which the element i occurred for the first time in the sequence. Then the solution for $n_i(t)$ starting from the initial condition $n_i(t_i) = 1$ is given by

$$n_i = \frac{t}{t_i}. \tag{5}$$

Now consider the cumulative distribution $P(n_i \leq n)$. From Eq. (5), we can write $P(n_i \leq n) = P(t_i \geq \frac{t}{n}) = 1 - P(t_i < \frac{t}{n})$. This leads to the estimate:

$$P(t_i < \frac{t}{n}) \simeq \frac{D(\frac{t}{n})}{D(t)} = n^{-\frac{\nu}{\rho}}. \tag{6}$$

2. $\nu > \rho$, when $D \simeq \frac{\nu-\rho}{a}t$. Again considering $t \gg N_0$, we write:

$$\frac{dn_i}{dt} \simeq \frac{\rho n_i}{(\rho + a\frac{\nu-\rho}{a})t} = \frac{\rho n_i}{\nu t}, \tag{7}$$

which yields the solution

$$n_i = \left(\frac{t}{t_i}\right)^{\frac{\rho}{\nu}}. \tag{8}$$

Proceeding as in the previous case, we find $P(n_i \leq n) = P(t_i \geq t\, n^{-\frac{\nu}{\rho}}) = 1 - P(t_i < t\, n^{-\frac{\nu}{\rho}})$, and thus

$$P(t_i < t\, n^{-\frac{\nu}{\rho}}) \simeq \frac{D(t\, n^{-\frac{\nu}{\rho}})}{D(t)} = n^{-\frac{\nu}{\rho}}, \tag{9}$$

obtaining the same functional expression of the asymptotic power-law behavior of the frequency-rank distribution as in the previous case.

The probability density function of the occurrences of the elements in the sequence is therefore $P(n) = \frac{\partial P(n_i < n)}{\partial n} \sim n^{-\left(1+\frac{\nu}{\rho}\right)}$, which corresponds to a frequency-rank distribution $f(R) \sim R^{-\frac{\rho}{\nu}}$.

Note that the estimates in equations (6) and (9) have been derived under the assumption that $t/n \gg 1$, i.e. in the tail of the frequency-rank distribution. In this respect, it is important to recognize that Zipf's and Heaps' laws are not trivially and automatically related, as is sometimes claimed. We certainly agree that Heaps' law can be derived from Zipf's law by the following random-sampling argument: if one assumes a strict power-law behaviour of the frequency-rank distribution $f(R) \sim R^{-\alpha}$ and constructs a sequence by randomly sampling from

this Zipf distribution $f(R)$, one recovers Heaps' law with the functional form $D(t) \sim t^{\beta}$ with $\beta = 1/\alpha$ [3, 4]. But the assumption of random sampling is strong and sometimes unrealistic. If one relaxes the hypothesis of random sampling from a power-law distribution, the relationship between Zipf's and Heaps' law becomes far from trivial. In our model, and in work by others [4], the relationship $\beta = 1/\alpha$ holds only asymptotically, i.e. only for large times, with α measured on the tail of the frequency-rank distribution.

In the main text we presented numerical results confirming the above analytical predictions for the first version of our model. Here we report numerical results for the second version of the model (employing the definition (ii.a)), summarized in the top-left panels of Fig. S0 and Fig. S1. The robustness of the results with respect to fluctuations of the model parameters ν and ρ was checked as follows. At each time step both ρ and ν were sampled from a uniform distribution (top-right), an exponential distribution (bottom-left) and a fat-tailed distribution with diverging variance, all with the same mean values $\bar{\rho} = 8$ and $\bar{\nu} = 5$. For the uniform distribution, ρ and ν were sampled from the intervals $[0, 2\bar{\rho}]$ and $[0, 2\bar{\nu}]$, while for the fat-tailed distribution, the chosen exponents were $\alpha_{\rho} = \frac{2\rho-1}{\rho-1}$ and $\alpha_{\nu} = \frac{2\nu-1}{\nu-1}$, which ensured the desired average values by choosing 1 as the minimum value.

In the case $\rho < \nu$ we recover the results of the well-known Yule-Simon Model (YSM) [5], originally proposed in the context of linguistics. In YSM, new words are added to a text (more generally a stream) with constant probability p at each time step, while with complementary probability $(1 - p)$, a word that has already occurred is chosen uniformly from within the text (or stream) generated so far. YSM leads to a Zipf's law with an exponent $-(1 - p)$ compatible with a linear growth in time of the number of different words. In the framework of our *urn model with triggering* we recover the same Zipf's exponents as well as the linear growth of $D(t)$ if $p = 1 - \frac{\rho}{\nu}$, with $\rho < \nu$[1]. The YSM is a paradigmatic example of a model that generates a fat-tail frequency-rank distribution $f(R) \sim R^{-\alpha}$ by using a rich-gets-richer mechanism. But it has the drawback that it does not reproduce both an $f(R)$ obeying a power-law behavior and a sublinear Heaps' exponent at the same time. Moreover, the YSM cannot reproduce values of α larger than 1 (which are found empirically in the frequency-rank distribution of words in certain texts). These problems were at the basis of the famous Simon-Mandelbrot dispute [6, 7, 8, 9, 10]. In our model the introduction of the parameter ν (describ-

[1]We note that if $\nu \gg 1$ when $a = \nu + 1$ (first version of the model) or $\nu \gg \rho$ and $\nu \gg 1$ when $a = \nu + 1 - \rho$ (second version of the model) our model also reproduces the same prefactor of the linear growth of $D(t)$ as in the YSM. This is evident by setting $a = \nu$ in Eq. (2).

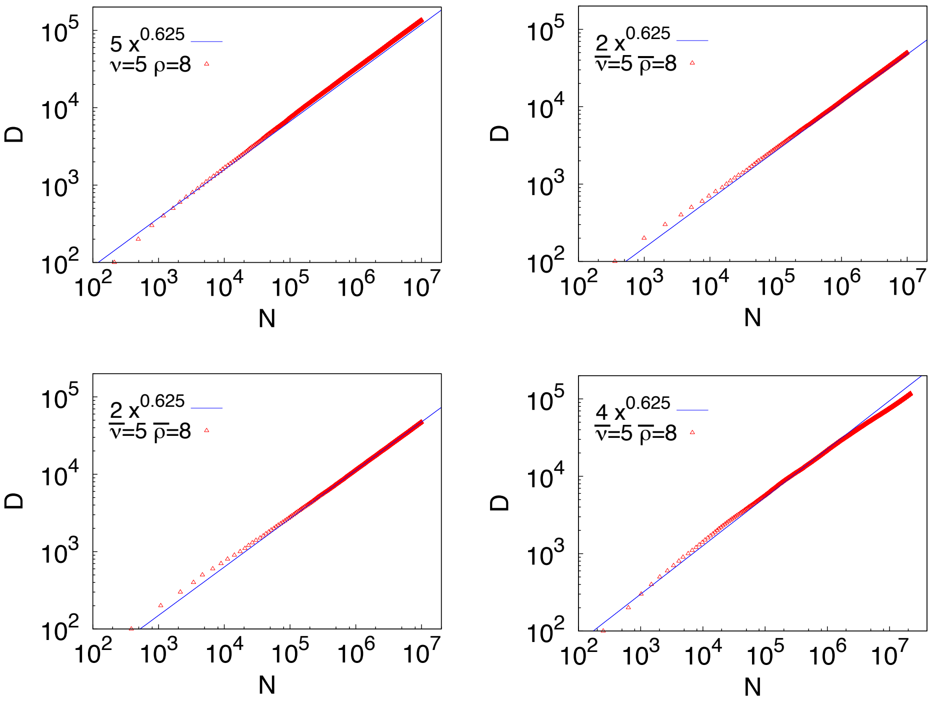

Figure S0: **Growth of the number of distinct elements (Heaps' law).** Top left: second version of the model without reinforcement on new words. Top right: original model with ρ and ν sampled from uniform distributions. Bottom left: original model with ρ and ν extracted from exponential distributions. Bottom right: original model with ρ and ν extracted from power law distributions. All distributions bear the same average values $\bar{\rho} = 8$ and $\bar{\nu} = 5$ (see text for details).

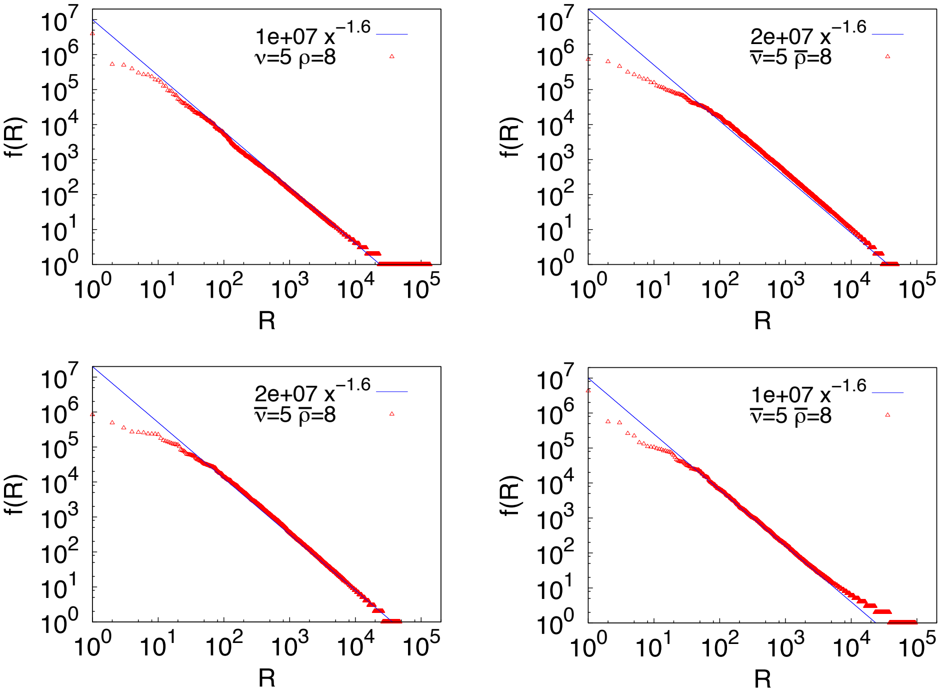

Figure S1: **Frequency-rank distribution (Zipf's law).** Top left: second version of the model without reinforcement on new words. Top right: original model with ρ and ν sampled from uniform distributions. Bottom left: original model with ρ and ν extracted from exponential distributions. Bottom right: original model with ρ and ν extracted from power law distributions. distributions bear the same average values $\bar{\rho} = 8$ and $\bar{\nu} = 5$. We have checked that the results do not depend on the initial condition N_0. This is set in all the simulations to the value $N_0 = 100$.

ing the expansion of the adjacent possible) heals these problems by confining the phenomenology of the YSM to the special case $\rho < \nu$.

1.3 Heaps' and Zipf's laws for the *urn model with semantic triggering*

We turn now to the counterparts of Heaps' and Zipf's laws for the *urn model with semantic triggering*. For the sake of completeness we recall the model's definition. One starts with an urn $\mathcal{U}$ with N_0 distinct elements, divided in $N_0/(\nu + 1)$ groups, the elements in the same group sharing a common label. After choosing the first element at random, the sequence $\mathcal{S}$ is constructed according to the following scheme:

(i) a weight 1 is given to: (a) each element in $\mathcal{U}$ with the same label, say A, as s_{t-1}, (b) to the element that triggered the enter in the urn of the elements with label A, and (c) to the elements triggered by s_{t-1}; a weight $\eta \leq 1$ is given to any other element in $\mathcal{U}$;

(ii) an element s_t is chosen from $\mathcal{U}$ with a probability proportional to its weight and appended to the sequence;

(iii) the element s_t is put back into $\mathcal{U}$ along with ρ additional copies of it;

(iv) if the chosen element s_t is new (i.e., it appears for the *first time* in the sequence $\mathcal{S}$) $\nu + 1$ brand new distinct elements, all with a common brand new label, are added to $\mathcal{U}$. These $\nu + 1$ new elements are given a weight $\eta = 1$ at the next time step $t + 1$ and each time the same mother element s_t is picked.

Note that if $\eta = 1$ this model corresponds to the simple urn model with triggering introduced earlier.

Figures S2 and S3 report numerical results for the Heaps' and Zipf's laws respectively, for some values of the parameters of the model ν, ρ and η. For this modified model with semantic triggering, the relation between the exponent β of the Heaps' law and the exponent $\alpha = 1/\beta$ of the Zipf's law continues to hold asymptotically, i.e. for large times, with α measured on the tail of the frequency-rank distribution. In particular, the time at which the above relation starts to hold depends on the exponent β of the Heaps' law. Larger times are needed for smaller β. The existence of a pre-asymptotic regime for the Zipf's law is observed also

in real datasets both for aggregated (see Fig. 1 of the main text) and for non-aggregated data (see the corresponding Section below). It is interesting to outline that this feature is captured only by the model with semantic triggering. This suggests that taking into account correlations is crucial to explain the apperance of different regimes in the statistics of real datasets.

We now outline the analysis leading to an estimate for the Heaps' exponent as a function of the model parameters ν, ρ and η. Observe that if we know the label of the last added element to the sequence $\mathcal{S}$, say s, we can write for the number of distinct elements $D(t)$ appearing in the sequence $\mathcal{S}$:

$$\frac{dD(t)}{dt} = \frac{N^s(t)}{N^s(t) + \eta N^{\bar{s}}(t)} \frac{N^s_D(t)}{N^s(t)} + \frac{\eta N^{\bar{s}}(t)}{N^s(t) + \eta N^{\bar{s}}(t)} \frac{N^{\bar{s}}_D(t)}{N^{\bar{s}}(t)} = \frac{N^s_D(t) + \eta N^{\bar{s}}_D(t)}{N^s(t) + \eta N^{\bar{s}}(t)} \tag{10}$$

where $N^s(t)$, $N^s_D(t)$, $N^{\bar{s}}(t)$ and $N^{\bar{s}}_D(t)$ denote respectively the number of elements with label s, the number of new (never used in the sequence $\mathcal{S}$) elements with label s, the number of elements with label different from s, and the number of new elements with label different from s, that are present in the reservoir $\mathcal{U}$ at time t.

The following relations hold:

$$\nu D(t) = N^s_D(t) + N^{\bar{s}}_D(t) \;\text{ and }\; U(t) = N^s(t) + N^{\bar{s}}(t), \tag{11}$$

where $U(t)$ is the number of total elements in the reservoir. It is worth remarking that if $\eta = 1$ one recovers Eq. (1).

We now drop the hypothesis of knowing the label of the last added element, and write a general equation for $D(t)$ of the form:

$$\frac{dD(t)}{dt} = \sum_k P(k) \frac{N^k_D(t) + \eta N^{\bar{k}}_D(t)}{N^k(t) + \eta N^{\bar{k}}(t)} = \sum_k P(k) \frac{N^k_D(t) + \eta(\nu D(t) - N^k_D(t))}{N^k(t) + \eta(U(t) - N^k(t))} \tag{12}$$

where the sum is over all the labels k present at time t in the reservoir $\mathcal{U}$ and $P(k)$ is the probability that the last added element to the sequence $\mathcal{S}$ at time t had the label k.

In order to close the equation (12), we should estimate $N^k(t)$ and $N^k_D(t)$ for a generic label k. Let us start by observing that $N^k_D(t) \leq \nu + 1$, and this term can be neglected in the large t limit with respect to $D(t)$.

We now leave the more complex problem of estimating $N^k(t)$ and we consider instead the probability $P(n)$ that $N^k(t) \equiv n$, substituting the sum over k in

equation (12) with the sum over the labels with the same number of occurrences n in the reservoir. We can thus write (asymptotically):

$$\frac{dD(t)}{dt} = \sum_n P(n) \frac{\eta\nu D(t)}{n(1-\eta) + \eta U(t)}. \tag{13}$$

We do not explicitly compute $P(n)$, but we consider two opposite limits:

1. We retain in the sum of equation (13) only the terms $n \simeq U(t)$. This approximation is sufficiently good when the frequency-rank distribution for the elements in $\mathcal{S}$ is sufficiently steep, corresponding to a high Zipf's exponent. Solving the equation (13) within this approximation, we obtain the result for the Heaps' exponent $\beta = \min(\frac{\nu\eta}{\rho}, 1)$.

2. When the probability $P(n)$ is large only for $n \ll U(t)$, we can neglect in the sum of equation (13) the term $n(1-\eta)$ with respect to $\eta U(t)$. Solving the equation (13) within this approximation, we obtain: $\beta \simeq \min(\frac{\nu}{\rho}, 1)$.

Summarizing, we have obtained lower and upper bounds for β: $\min(\frac{\nu\eta}{\rho}, 1) \leq \beta \leq \min(\frac{\nu}{\rho}, 1)$, that are satisfied by the simulation results shown in Figs. S2 and S3 .

2 The random walk model for the dynamics of novelties

Our urn model with triggering, both with and without semantics, can be mapped in the framework of the exploration of an evolving graph $\mathcal{G}$ through a random walker (RW). In particular, the RW dynamics can be constructed as follows (see also figure S5).

We start with a graph $\mathcal{G}$ of N_0 nodes, divided in $N_0/(\nu+1)$ cliques, each node in the same clique sharing a common label. We then draw a link between each pair of nodes belonging to different cliques with probability $\eta \leq 1$. Starting with the RW in a random position, and with a weight $w_j = 1$ for each node j, at each time step:

(i) move the RW to a neighbour node or keep it on the present node (self-loops allowed) with a weight-dependent probability;

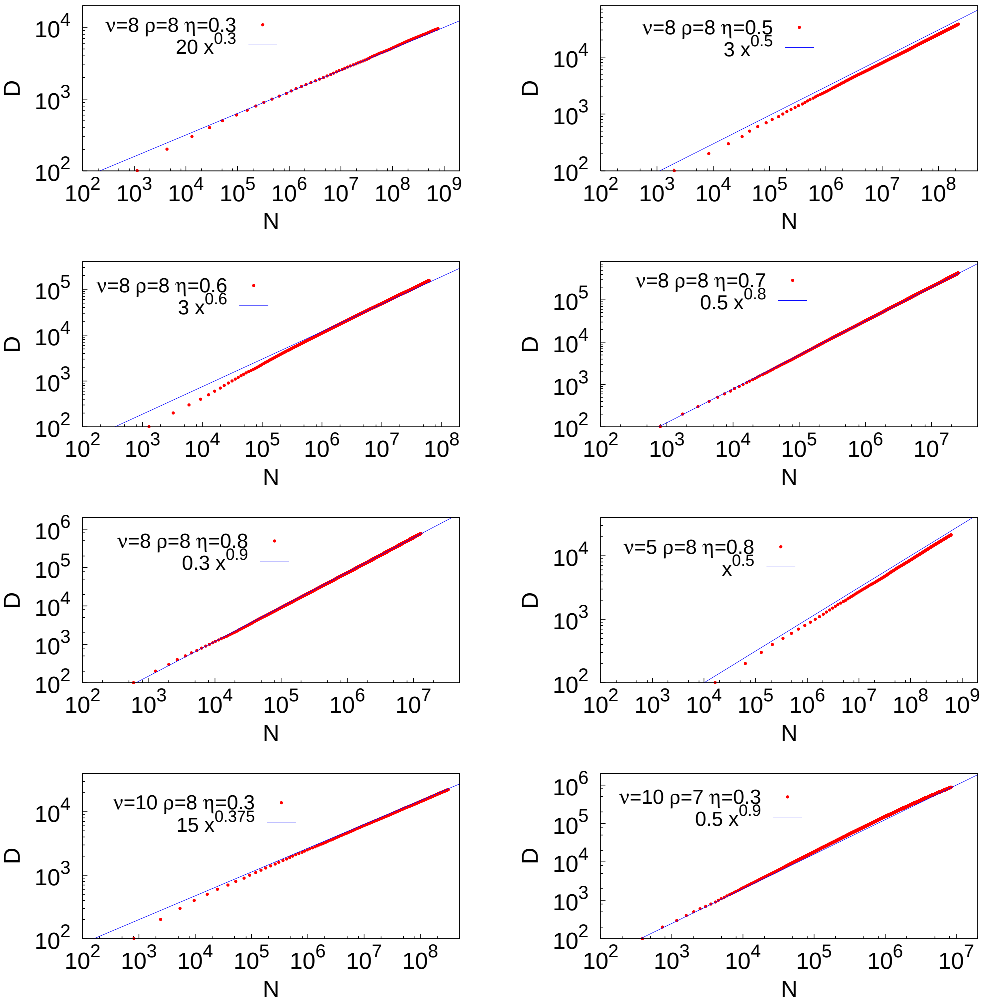

Figure S2: **Growth of the number of distinct elements (Heaps' law).** Heaps' law for several values of the parameters of the urn model with semantic triggering. Straight lines show functions of the form ax^{β}, where a is a constant. In all the simulations $N_0 = \nu+1$. The observed exponents are within the theoretical bounds $\min(\frac{\nu\eta}{\rho}, 1) \leq \beta \leq \min(\frac{\nu}{\rho}, 1)$.

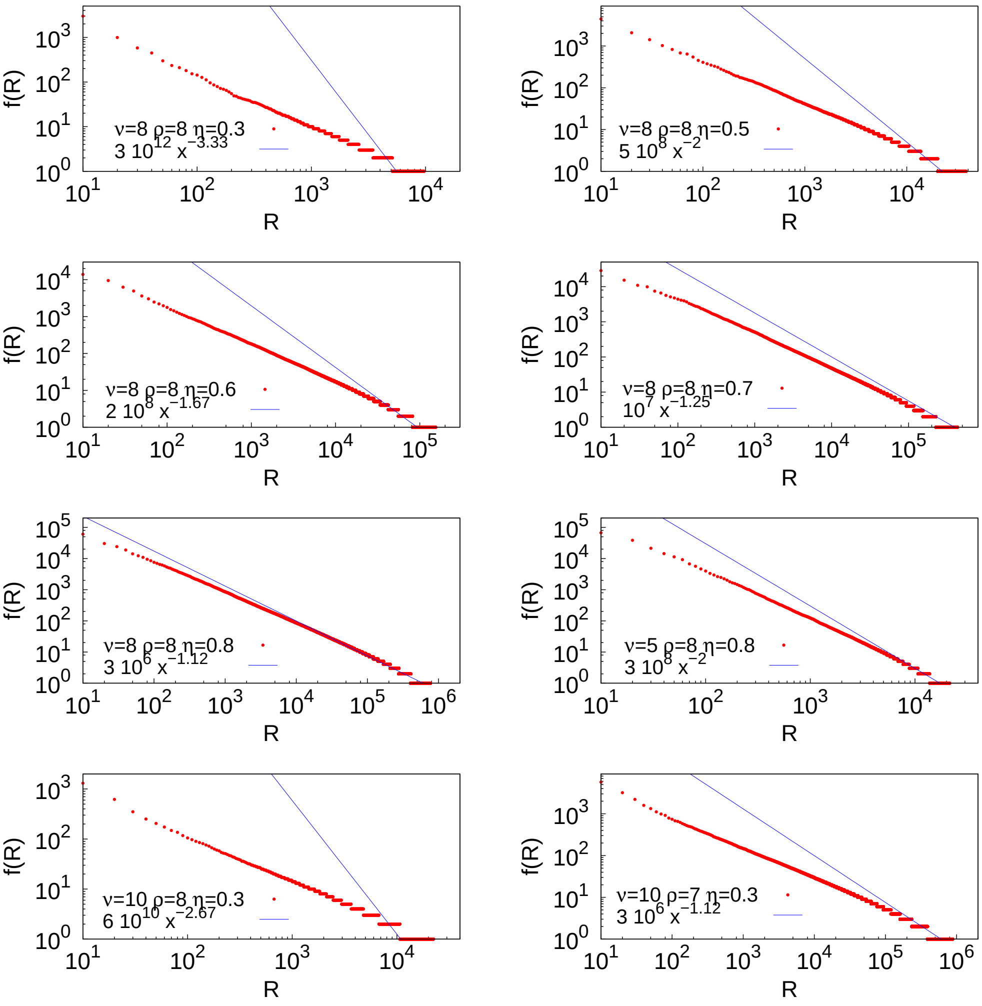

Figure S3: **Frequency-rank distribution (Zipf's law).** Zipf's law for several values of the parameters of the urn model with semantic triggering. The exponent α of the tail of the distributions is compatible with the exponent β of the Heaps' law. It is worth remarking how the correspondence gets worst when the exponent of the Heaps' law decreases, since in this case one needs extremely longer simulations in order to get sufficient statistics on the tail. Straight lines show functions of the form $ax^{-1/\beta}$, where a is a constant. In all the simulations $N_0 = \nu + 1$.

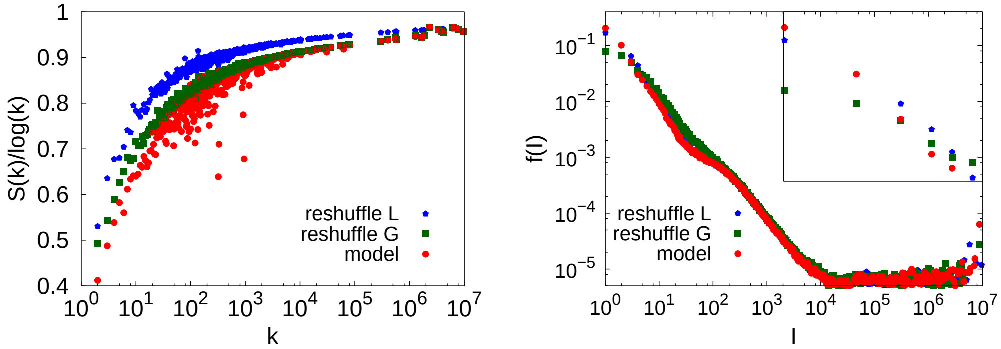

Figure S4: **Entropy (left) and intervals (right) distribution in the random walk model mapping the urn model with semantic triggering**. Left: Entropy of a sequence associated to a specific label A vs. the number of events, k, with that label. The entropy is averaged for each k over the labels with the same number of occurrences. The plot shows an average over 10 realizations of the process with parameters values: $\nu = 10$, $\rho = 7$, $\eta = 0.2$, and $N_0 = \nu + 1$, corresponding to a Heaps' exponent of $\beta = 0.29$ (see figure S6). In each realization the sequence $\mathcal{S}$ has length $N = 10^7$. Right: Results for the time intervals distribution for the same data as for the entropy. The color code is red for the actual sequence, green for the global reshuffle of the sequence $\mathcal{S}$, and blue for the local reshuffle (see text). In the inset a zoom of the first intervals' lengths is shown.

(ii) reinforce the selected node weight $w_i \to w_i + \rho$;

(iii) if the node visited is new (i.e., it is visited for the *first time*) add a clique with $\nu + 1$ new nodes connected to the just visited node, each node in the new clique sharing a common label, different from all the preexisting ones. In addition draw a link between each node in the newly added clique and all the preexisting nodes of the network with probability η.

If $\eta = 1$ this model maps one-to-one to the urn model with triggering introduced in the main text. When $\eta < 1$ the correspondence with the *urn model with semantic triggering* is not one-to-one: in the case of the graph the connections between two nodes are fixed (or *quenched*), i.e. either they are there or they are not, whether the possibility of going from one element to each of the others in the urn model is always probabilistic (one can imagine that this corresponds to an *annealed* version of the graph model, where links are continuously re-drawn according to a fixed probability). Despite this difference, the statistical properties of the two models

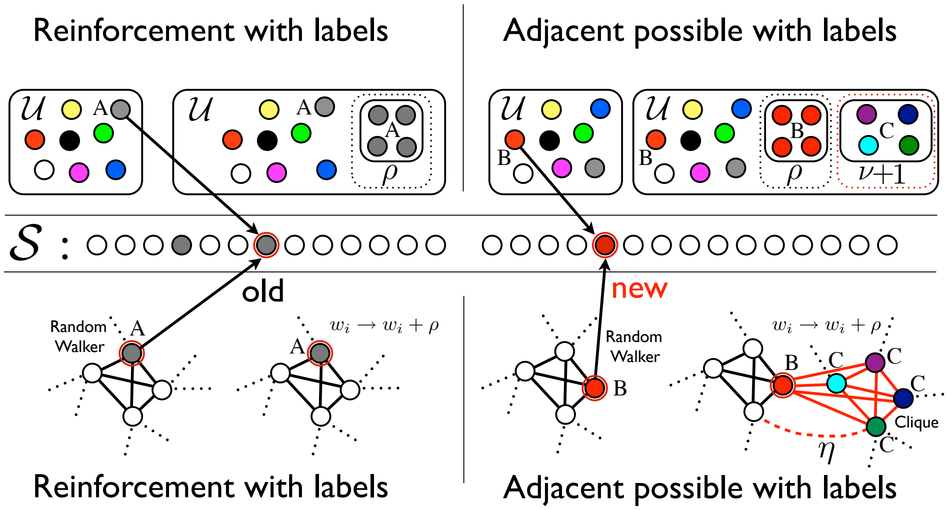

Figure S5: **Models** *Top*: scheme of the *urn model with semantic triggering*. On the left panel we describe a generic reinforcement step of the dynamics, where one element already drawn earlier on time is drawn from the urn $\mathcal{U}$ (the gray ball). In this case one adds this element to $\mathcal{S}$ (depicted at the center of the figure) and, at the same time, put ρ additional gray balls to $\mathcal{U}$, all with the same label A of the parent gray ball. On the right panel we illustrate a generic adjacent possible step of the dynamics. Here, upon drawing a new ball (red) from $\mathcal{U}$, $\nu + 1$ brand new balls are added to $\mathcal{U}$, all sharing a brand new label C, along as the ρ red balls of the reinforcement step that takes place at each time step. *Bottom*: scheme of the random walk (RW) based model for the dynamics of novelties. Whenever a RW visits an already visited node (gray node on the left panel) one adds a gray element to $\mathcal{S}$ and reinforce the node's weight according to the formula $w_i \to w_i + \rho$. Whenever the RW visits for the first time a node i (red node in the right panel), a new clique (representing the newly created adjacent possible) with $\nu + 1$ nodes is added to the graph, all the nodes sharing a brand new label C. Each node of the clique is connected to the red node, and with a probability η to the other already existing nodes. At the same time one adds the red element to $\mathcal{S}$, always reinforcing the node's weight according to the formula $w_i \to w_i + \rho$.

turn out to be equivalent from a qualitative point of view also in the case $\eta < 1$. In figure S6 we report some examples of the Heaps' and Zipf's laws for the RW model, for different values of the parameters ν, ρ and η, while in figure S4 we give an example of the triggering events as measured by the entropy S associated to the labels and the distribution $f(l)$ of triggering time intervals between two successive appearance in the sequence $\mathcal{S}$ of the same label (see Section Methods in the main text).

As a final remark, we note that the RW modeling scheme allows one to more naturally extend the structure of the semantic relations between the different elements. The semantic relations are in fact encoded in the growing graph topology, and one can imagine different ways of linking the new nodes, corresponding to more complex and realistic semantic structures.

3 Details of the datasets used

3.1 Gutenberg Corpus

The corpus of English texts used in the analysis was collected by a crawl of the material available at the Gutenberg Project ebook collection [11]. The crawl was carried on February 2007 and resulted in a set of about 7500 non-copyrighted ebooks in plain ASCII format. After a filtering procedure used to remove from the analysis all non-English texts, we came up with ca. 4600 texts, dealing with diverse subjects and including both prose and poetry. In total, the corpus consisted of about 2.8×10^8 words, with about 5.5×10^5 different words. In the analysis we ignored capitalization. Words sharing the same lexical root were considered as different, i.e., the word *tree* was considered different from *trees*. Homonyms, as for example the verbal past perfect *saw* and the substantive *saw*, were treated as the same word. The aggregated analysis is performed by putting all the books in a random order one after the other in a single text. The texts used in the non aggregated analysis are listed in Table S1.

3.2 Delicious

Delicious [12] is an online social annotation platform of bookmarking where users associate keywords (tags) to web resources (URLs) in a post, in order to ease the process of their retrieval. The dataset used for the present analysis [13] consists of approximately 5×10^6 posts, comprising about 650,000 users, 1.9×10^6 resources

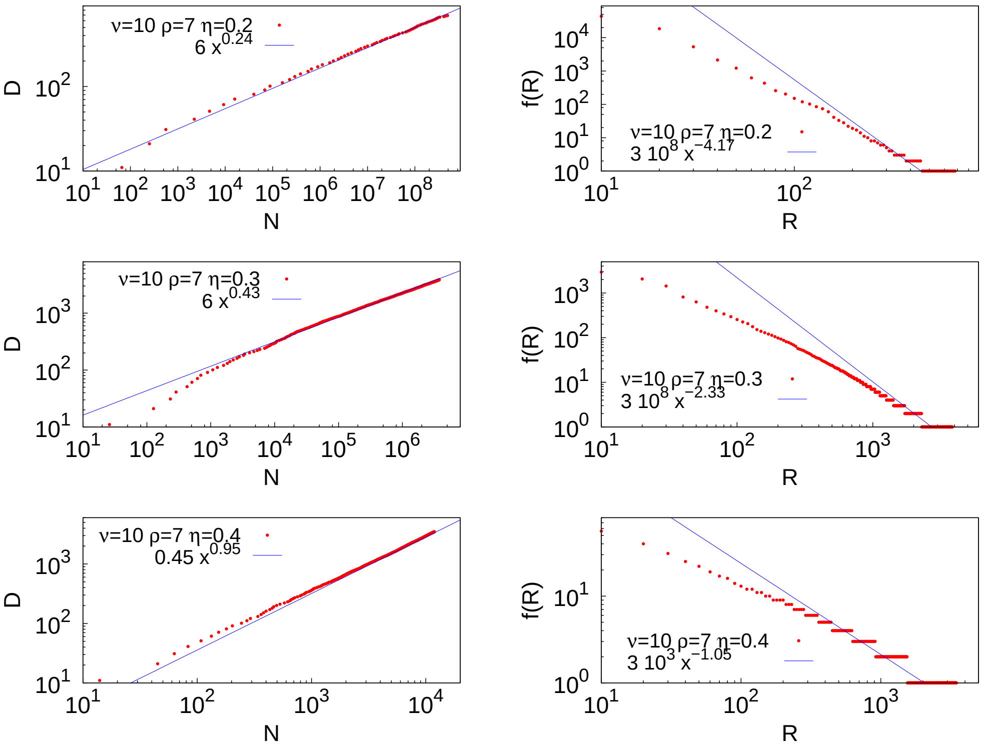

Figure S6: **Growth of the number of distinct elements (Heaps' law) and frequency-rank distribution (Zipf's law) for the RW model.** Left: Heaps' law for several values of the parameters of the random walk model mapping the urn model with semantic triggering. Straight lines show functions of the form ax^{β}, where a is a constant. Right: Zipf's law for the corresponding values of the parameters of the random walk model. The exponent α of the tail of the distributions is compatible with the exponent β of the Heaps' law. Straight lines show functions of the form $ax^{-1/\beta}$, where a is a constant. In all the simulations $N_0 = \nu + 1$.

Author	Work	Total Nr of words	Nr of distinct words	α	β
C. Dickens	Hard Times	124109	8747	1.17	0.58
C. Dickens	David Copperfield	426904	14026	1.43	0.53
C. Dickens	Oliver Twist	191395	10177	1.30	0.55
H. Melville	Moby-Dick	252571	17136	1.22	0.60
S. Butler	Odyssey (prose)	131444	6363	1.51	0.50
A. Pope	Odyssey (verse)	132461	8292	1.37	0.50
Homer	Odyssey	86868	17506	1.03	0.70
Homer	Iliad	112082	21853	1.05	0.68

Table S1: **Texts from the Gutenberg site used in the non-aggregated analysis.** For each text we report the total number of words, total number of *distinct* words and the estimated values of the (minus) the Zipf's exponent and Heaps' exponent. Note that $1/\alpha > \beta$ since the single texts are not sufficiently long to allow the asymptotic regime to be visible, and the frequency-rank distribution curve has not yet gone through the crossover visible around $10^4 \sim 10^5$ in the analogous curve of the whole Gutenberg dataset, showed in the main article.

and 2.5×10^6 distinct tags (for a total of about 1.4×10^8 tags), and covering almost 3 years of user activity, from early 2004 up to November 2006. Since *Delicious* is case-preserving but not case sensitive, we ignored capitalization in tag comparison, and counted all different capitalization of a given tag as instances of the same lower-case tag. The time stamp of each post was used to establish post ordering and determine the temporal evolution of the system.

In the non-aggregated analysis we extracted from the Delicious dataset the posts of the three most active users (RangerRick, hidekii, PeterPeter) and two random ones (Vitelot, AndreaB).

3.3 Last.fm

Last.fm [14] is a music website equipped with a music recommender system. Last.fm builds a detailed profile of each user's musical taste by recording details of the songs the user listens to, either from Internet radio stations, or the user's computer or many portable music devices. The data set we used [15, 16] contains the whole listening habits of 1000 users till May, 5th 2009, recorded in plain text form. It contains about 1.9×10^7 listened tracks with information on user, time stamp, artist, track-id and track name.

For the non-aggregated analysis we consider only the data of the five most active listeners.

3.4 English Wikipedia

The English Wikipedia database we analyzed consists of 323 compressed files summing up to a total of 48 GB of disk space. The uncompressed overall size is around 20 TB. The Wikipedia database we collected [17], dates back to March 7th, 2012.
Due to the database huge dimension, we had to develop a special procedure to extract the information we needed. The computer we used to process the database is a multi-core machine mounting 8 Intel(R) Xeon(R) X3470 CPU, with a 2.93 GHz working clock frequency, with a RAM of 16 GB.
The database contains a copy of all pages with all their edits in plain text by using the XML structure.

In order to perform the analysis related to the detection of triggering events, we extracted from the database the following information. First of all, we identified for each new born page, say B, the page, say A, that internally linked the new born page for the first time. We call the page A the *mother page* of B and we identify for each edit its mother page as its label (note that several edits can have the same mother page, i.e., the same label). We then follow the steps below:

(1) To each edit event we associate: (i) the wikipedia page exclusive identification number (ID), (ii) the user (wikipedia contributor) ID (UID), (iii) the edit ID (EID), (iv) its time stamp (TS), (v) the PID of its mother page;

(2) from the list of all edits endowed with the information discussed in (1), we removed the multiple edits of the same page done by the same user, retaining his/her first edit;

(3) we sorted the list (2) according to increasing time stamp.

For the non-aggregated analysis we focused on seven randomly chosen editors. Special care was needed to understand whether a selected user was human. In fact, the most active editors of Wikipedia are robots performing minor changes routinely.

4 Results for non aggregated data

The analysis performed in the main text, involving the previously described datasets as a whole, is here repeated for some of their selected records. In case of the Gutenberg dataset, we chose texts; in Wikipedia, Last.fm and Delicious, we chose editors, listeners and tagging users respectively.

User ID	Total Nr of edits	Nr of distinct edits	α
1188594	14613	8619	0.45
1638938	6776	3094	0.56
23958	19226	7295	0.70
281454	1480	974	0.41
2829979	11642	4622	0.50
356300	10415	3738	0.83
62662	6118	975	1.06
82835	937852	716418	0.41
99037	128802	78961	0.57

Table S2: **Editors of Wikipedia used in the non-aggregated analysis.** For each editor we report: the total number of edited articles; the total number of *distinct* edited articles; the observed values α of the (minus) the Zipf's exponent. The values of the Heaps' exponent for all the considered users turn out to be $\beta \simeq 1$, in agreement with the alpha values $\alpha \leq 1$ as predicted by the model.

Heaps' and Zipf's law

The analysis of Heaps' law is displayed in Fig. S7 and shows an asymptotic sublinear power-law behaviour in the case of texts (see Table S1) and a possible linear behavior for Wikipedia editors (see Table S2). In the case of Last.fm and Delicious, the sublinear behavior can still be spotted but the dictionary curves are less smooth than those of Wikipedia and Gutenberg. The reason is that in both Last.fm and Delicious, users may import large blocks of music tracks and website bookmarks from their local storage, thus introducing a sort of discontinuity in time. This discontinuity is obviously less appreciable in figure S8, were we show the frequency-rank distribution of words in selected texts, lyrics in selected listeners using Last.fm, wiki-articles for selected editors in Wikipedia and tags for selected users of Delicious. In fact, the frequency-rank is insensible to the temporal ordering of the elements, being a global statistical property of the sample. Note how the more inflected ancient Greek language results in a smaller Zipf's exponent than that of English texts and correspondingly in a larger Heaps' exponent (see Table S1). It is also worth noting that the measured exponent β of the Heaps' law in the selected texts does not happen to be the reciprocal of the measured Zipf's exponent α. In the main text we have shown that the frequency-rank curve of the whole Gutenberg corpus displayed two main behaviors with different exponents (an analogous observation was shown in Ref. [18]) so that, when inferring α from texts containing $10^4 \sim 10^5$ distinct words, one tends to underestimate it. The Heaps' law, instead, is already sufficiently sensible to sample the tail of

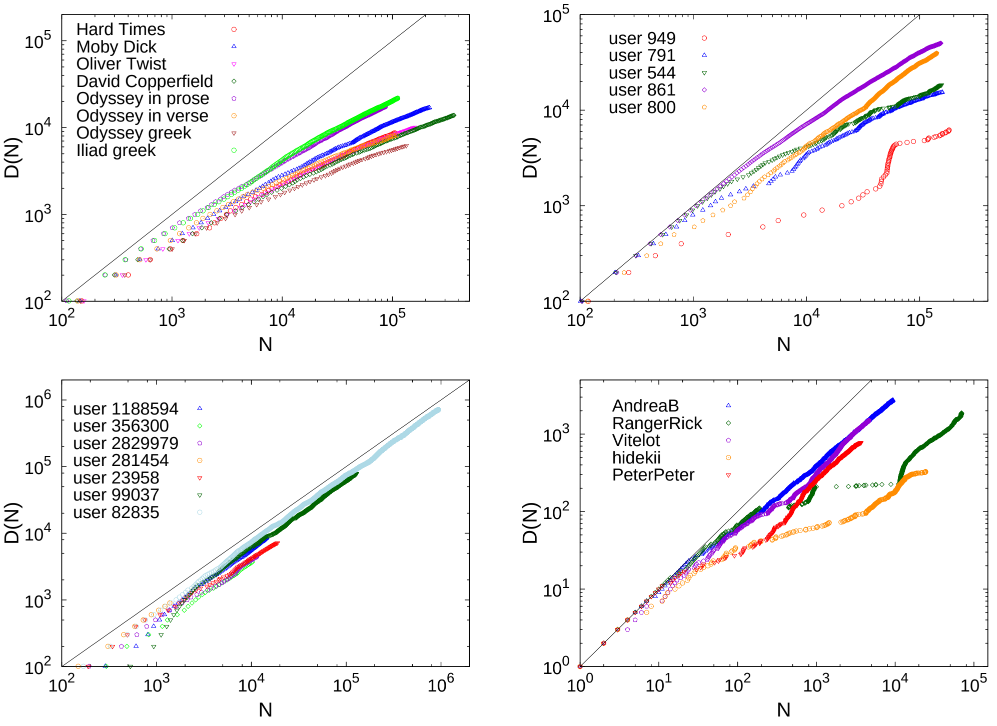

Figure S7: **Growth of the number of distinct elements (Heaps' law).** Top-left: Selected masterpieces from the Gutenberg dataset (words as elements); Top-right: most active users in Last.fm (lyrics as elements); Bottom-left: selected (human) random editors of Wikipedia with appreciable activity (wiki-articles as elements); Bottom-right: Selected users of Delicious (tags as elements). The linear growth is indicated by the straight line. The discontinuities in both right panels can be ascribed to a data import from other sources (local playlists to Last.fm, local bookmarks to Delicious).

the distribution so that the measured α and β are such that $1/\alpha > \beta$.

It is interesting to observe that the asymptotic validity of the relation between the Zipf's and Heaps' exponents is also captured by our model with semantic triggering. Fig.s S1 and S6 display the asymptotic correspondence $\beta = 1/\alpha$ along as the existence of at least another regime at lower ranks whose extension depends on the combination of parameters ν, ρ and η.

Another feature is worth to be mentioned. By looking at Fig. S7 we find that the growth of the number of distinct article edited in Wikipedia by users is linear. Our Polya's urn model accounts for this possibility as well, by predicting a connection between the Zipf's exponent and the slope of the linear dictionary growth.

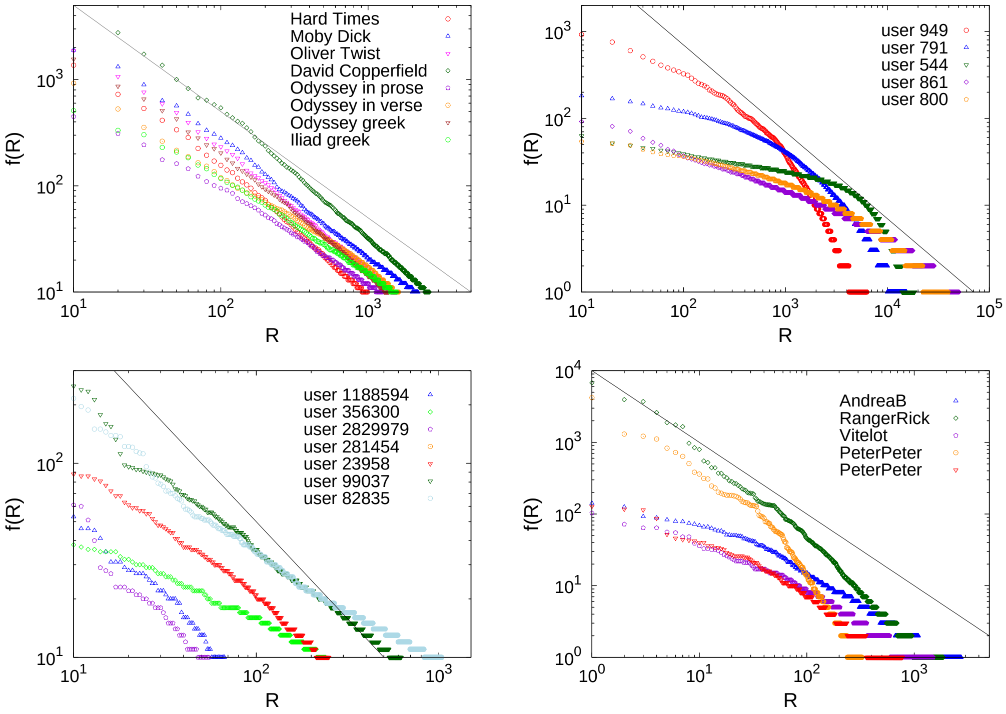

Figure S8: **Frequency-rank distribution (Zipf's law).** Top-left: Selected masterpieces from the Gutenberg dataset (words as elements); Top-right: most active users in Last.fm (lyrics as elements); Bottom-left: selected (human) random editors of Wikipedia with appreciable activity (wiki-articles as elements); Bottom-right: Selected users of Delicious (tags as elements). The straight line shows the strict Zipf's law with $\alpha = 1$ as a guide for the eye.

Triggering events

To detect whether in a sequence there is a triggering mechanism in play, we make use of the definition of entropy (see Eq. 2 of the main text) and look at the distribution of time intervals between elements of the same class (see Section Methods in the main text).

For example, when listening to a certain lyric of a given artist, we could be tempted to listen to other of her lyrics. In that case, the occurrences of the lyrics' artist will be clusterized in the sequence more than an uncorrelated poissonian process. At the same time, we expect that the distribution of time intervals between the lyrics of the same artist will be more biased toward small time intervals than a poissonian process. In the case of lyrics, the class of elements is given by their artist, in Wikipedia by the wiki-article (*mother page*) that first linked to a new wiki-page, while in texts we considered each word as bearing its own class, lacking of a satisfactory classification of words in semantic areas.

In order to distinguish between sequences ruled by a random poissonian process from sequences featuring triggering events, we show (we already reported the corresponding results for Gutenberg texts in the main text) in figures S9 and S10 the entropy and interval distribution curves of selected Last.fm listeners and wiki editors (red dots), together with the correspondingly randomly shuffled sequences (blue dots) and the *locally* shuffled sequences (green dots). The latter are achieved by shuffling the subsequence that goes from the element following the first occurrence of a given element, to the end. These figures confirm that also at the user level one obtains the same results of the whole datasets. In particular, the drop of the entropy around the value of 10 in the three selected Last.fm listeners can be a consequence of the typical number of songs in a song album: who listens one song of an album, tends to browse all of it, so that a dozen of songs with the same artist appear heavily clusterized at short times, thus dropping the associated entropy value.

The interest of looking at triggering events on single books (we already reported about individual texts of the Gutenberg corpus in the main text), or considering a single contributor of Wikipedia or a single Last.fm user is to investigate the nature of the correlations observed in the whole databases. In particular, the question is whether the statistical signatures we detected emerge as an effect of a collective process or are present also at the single user level. The results reported in figures S9 and S10 show that the adjacent possible mechanism plays a role also on the individual level, and its effect is enhanced in collective processes.

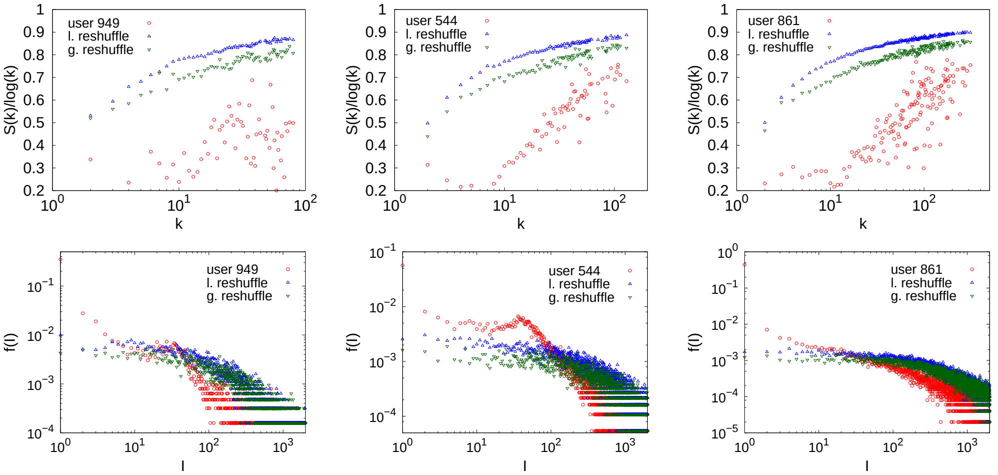

Figure S9: **Triggering events for single users in the Last.fm dataset.** Top: normalized average entropy in selected listeners (red dot) and in the locally (blue dots) an globally (green dots) reshuffled playlists. Lower values of the entropy correspond to higher clusterized occurrences of elements. Bottom: Time intervals distribution. More clusterized data result in higher values of the distribution at low interval lengths.

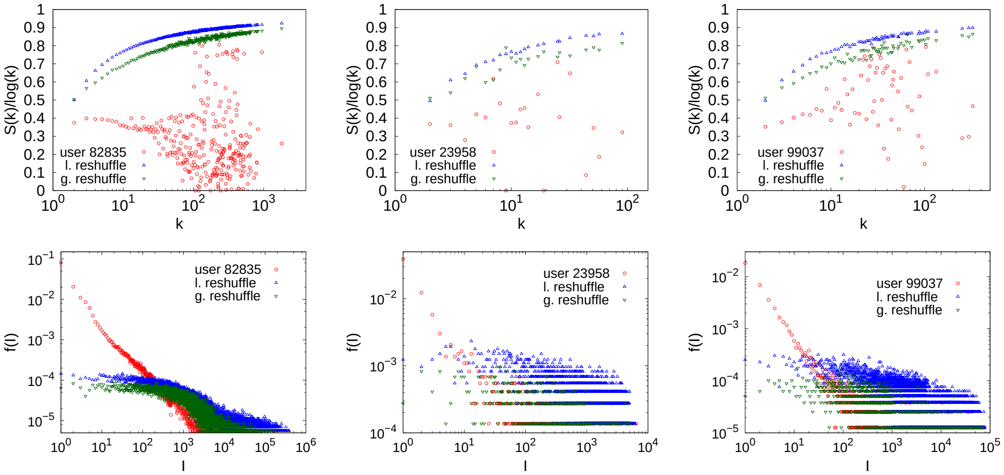

Figure S10: **Triggering events for single users in the Wikipedia dataset.** Top: normalized average entropy in selected editors (red dot) and in the locally (blue dots) an globally (green dots) reshuffled wiki-articles. Lower values of the entropy correspond to higher clusterized occurrences of elements. Bottom: Time intervals distribution. More clusterized data result in higher values of the distribution at low interval lengths.

References

[1] Zipf, G. K. *Human Behavior and the Principle of Least Effort* (Addison-Wesley, Reading MA (USA), 1949).

[2] Mahmoud, H. *Pólya Urn Models*. Texts in Statistical Science series (Taylor and Francis Ltd, Hoboken, NJ, 2008).

[3] Serrano, M. A., Flammini, A. & Menczer, F. Modeling statistical properties of written text. *PLoS ONE* **4**, e5372 (2009).

[4] Lü, L., Zhang, Z.-K. & Zhou, T. Zipf's law leads to Heaps' law: Analyzing their relation in finite-size systems. *PLoS ONE* **5**, e14139 (2010).

[5] Simon, H. A. On a class of skew distribution functions. *Biometrika* **42**, 425–440 (1955).

[6] Mandelbrot, B. A note on a class of skew distribution functions. analysis and critique of a paper by H. Simon. *Information and Control* **2**, 90 (1959).

[7] Simon, H. A. Some further notes on a class of skew distribution functions. *Information and Control* **3**, 80 (1960).

[8] Mandelbrot, B. Final note on a class of skew distribution functions: Analysis and critique of a model due to H. A. Simon. *Information and Control* **4**, 198–216 (1961).

[9] Simon, H. A. Reply to ”final note” by Benoit Mandelbrot. *Information and Control* **4**, 217–223 (1961).

[10] Mandelbrot, B. Post scriptum to “final note”. *Information and Control* **4**, 300–304 (1961).

[11] Hart, M. Project Gutenberg (1971). URL `http://www.gutenberg.org/`.

[12] Schachter, J. del.icio.us (2003). URL `http://delicious.com/`.

[13] Cattuto, C., Baldassarri, A., Servedio, V. D. P. & Loreto, V. Vocabulary growth in collaborative tagging systems. *Arxiv preprint arXiv: 0704.3316* (2007).

[14] Last.fm (2002). URL http://last.fm.

[15] Music recommendation datasets for research (2010). URL http://www.dtic.upf.edu/ ocelma/MusicRecommendationDataset/index.html.

[16] Celma, O. *Music Recommendation and Discovery in the Long Tail* (Springer, 2010).

[17] http://dumps.wikipedia.org/enwiki/20120307/ (2012).

[18] Montemurro, M. A. Beyond the ZipfMandelbrot law in quantitative linguistics. *Physica A* **300**, 567–578 (2001).
